# Barriers to and determinants of the use of intermittent preventive treatment of malaria in pregnancy in Cross River State, Nigeria: a cross-sectional study

**DOI:** 10.1186/s12884-016-0883-2

**Published:** 2016-05-04

**Authors:** Soter Ameh, Eme Owoaje, Angela Oyo-Ita, Caroline W. Kabiru, Obaji E. O. Akpet, Aniekan Etokidem, Okokon Enembe, Nnette Ekpenyong

**Affiliations:** Department of Community Medicine, College of Medical Sciences, University of Calabar, Calabar, Cross River State Nigeria; Medical Research Council/Wits Rural Public Health and Health Transitions Research Unit (Agincourt), School of Public Health, Faculty of Health Sciences, University of the Witwatersrand, Johannesburg, South Africa; Department of Community Medicine, College of Medicine, University of Ibadan, Ibadan, Oyo State Nigeria; African Population and Health Research Center, Nairobi, Kenya

**Keywords:** Determinants, Barriers, Use, Sulfadoxine-pyrimethamine, Intermittent preventive treatment of malaria in pregnancy, Cross River State, Nigeria

## Abstract

**Background:**

Malaria in pregnancy (MIP) has serious consequences for the woman, unborn child and newborn. The use of sulfadoxine-pyrimethamine for the intermittent preventive treatment of malaria in pregnancy (SP-IPTp) is low in malaria endemic areas, including some regions of Nigeria. However, little is known about pregnant women’s compliance with the SP-IPTp national guidelines in primary health care (PHC) facilities in the south-south region of Nigeria. The aim of this study was to identify the barriers to and determinants of the use of SP-IPTp among pregnant women attending ANC in PHC facilities in Cross River State, south-south region of Nigeria.

**Methods:**

A cross-sectional survey was conducted in 2011 among 400 ANC attendees aged 15–49 years recruited through multistage sampling. Binary logistic regression was used to determine the factors associated with the use of SP-IPTp in the study population.

**Results:**

Use of SP-IPTp was self-reported by 41 % of the total respondents. Lack of autonomy in the households to receive sulfadoxine-pyrimethamine (SP) during ANC was the main barrier to use of IPTp (83 %). Other barriers were stock-outs of free SP (33 %) and poor supervision of SP ingestion by directly observed treatment among those who obtained SP from ANC clinics (36/110 = 33 %). In the multivariate logistic regression, the odds of using SP-IPTp was increased by the knowledge of the use of insecticide treated nets (ITNs) (OR = 2.13, 95 % CI: 1.70–3.73) and SP (OR = 22.13, 95 % CI: 8.10–43.20) for the prevention of MIP. Use of ITNs also increased the odds of using SP-IPTp (OR = 2.38, 95 % CI: 1.24–12.31).

**Conclusions:**

Use of SP-IPTp was low and was associated with knowledge of the use of ITNs and SP as well as the use of ITNs for the prevention of MIP. There is a need to strengthen PHC systems and address barriers to the usage of SP-IPTp in order to reduce the burden of MIP.

**Electronic supplementary material:**

The online version of this article (doi:10.1186/s12884-016-0883-2) contains supplementary material, which is available to authorized users.

## Background

Remarkable progress has been made in the global fight against malaria. However, 3.4 billion people, including pregnant women, are still at risk of malaria [1]. The brunt of the global malaria burden is borne by sub-Saharan Africa (SSA) [1, 2], where over 30 million women become pregnant annually in malaria endemic areas [2]. Pregnant women are the main adult group at risk for malaria infection in endemic areas in SSA [2]. In Nigeria, nearly 110 million cases of malaria are clinically diagnosed per year. This makes malaria the most common cause of hospital attendance in all age groups, with estimated annual economic loses of over US$ 835 million from cost of treatment and absenteeism from work and school [3–5]. It is estimated that malaria is responsible for about 11 % of overall maternal mortality in Nigeria [4, 5].

Malaria in pregnancy (MIP) can have serious health consequences for the woman, unborn child and newborn. The direct effect of MIP on the mother is severe anaemia, resulting in an increased risk of maternal mortality. The indirect consequences of MIP are twofold: (i) intrauterine death/growth retardation of the foetus and (ii) low birth weight in the newborn with consequent higher risks of infant mortality and impaired child development [2].

A three-prong approach is recommended for the control of MIP in SSA: use of sulfadoxine-pyrimethamine for the intermittent preventive treatment of malaria in pregnancy (SP-IPTp), use of insecticide-treated bed nets (ITNs), and effective case management of malarial illness [2].

About 90 % of pregnant women in Nigeria attend some form of antenatal care (ANC) service [4]. This offers an immense opportunity to encourage women to utilise IPTp during ANC visits [4], particularly in Primary Health Care (PHC) facilities, which are the entry point into Nigeria’s health care system. In 2005, the Federal Ministry of Health (FMOH) in Nigeria adopted the IPTp as a part of focused ANC [6]. Provision of SP, at no cost to recipients, through Directly Observed Treatment (DOT) supervised by a skilled healthcare provider in public and faith based/NGO antenatal facilities is one of the strategies used to achieve the target of 90 % of pregnant women receiving at least two doses of sulfadoxine-pyrimethamine (SP) in the second and third trimesters of pregnancy [4].

The current World Health Organization (WHO) IPTp guidelines require that the first dose of SP-IPTp be given as early as possible in the second trimester of gestation with subsequent doses given at least one month apart. The last dose can be administered up to the time of delivery without safety concerns [7].

According to the 2009 Nigeria Health and Demographic Survey (NHDS), 8 % of pregnant women reported the use of one dose of SP-IPTp [8]. Studies have reported low use of SP-IPTp in various regions of Nigeria [9–16] and SSA [17]. Some of the perceived barriers to SP-IPTp use include drug stock-outs in the health facilities, lack of provider knowledge of the IPTp protocol, women’s belief that SP is harmful to the foetus, and low levels of awareness of the use of IPTp as a malaria preventive measure [10, 15, 17, 18]. Various factors have been identified as predictors of SP-IPTp use in PHC facilities in different regions of Nigeria, all of which vary in seasonality, intensity and duration of malaria transmission [19]. Knowledge of prophylaxis for malaria prevention is associated with SP-IPTp use in south-west Nigeria [20, 21], while advanced maternal age, higher education, higher parity, lower gestational age at registration for ANC, and use of ITNs are associated with use of SP-IPTp in northern Nigeria [14]. However, little is known about the determinants of the use of free SP-IPTp in accordance with the national treatment guidelines among women utilising ANC services in PHC facilities in south-south Nigeria. The aim of this research was to identify the barriers to and determinants of the use of free SP-IPTp by pregnant women attending ANC clinics in PHC facilities in Cross River State, south-south Nigeria.

## Methods

### Study design and settings

We conducted a cross-sectional study to assess the use of IPTp by pregnant women attending ANC clinics in PHC facilities in Cross River State between September and December 2011. The total population of the state is 2,892,988: males - 50.9 % (1,471,967) and females - 49.1 % (1,421,021) [22]. There are 18 Local Government Areas (LGAs) in Cross River State. Of these, five, six and seven LGAs constitute the northern, central and southern senatorial or political districts that make up the state, respectively. The state is situated in the tropical rain forest belt of Nigeria where malaria transmission is perennial [19].

### Political and health structure

Nigeria is divided into six geo-political regions (north-east, north-west, north-central, south-east, south-west and south-south) to better enhance political administration of the country by the Federal Government [23]. The seasonality, intensity and duration of malaria transmission vary across these geo-political regions of Nigeria with the duration of malaria season being perennial in most of the southern regions, but lasting three months or less in the northern regions [19].

Three levels of health care delivery exist in Nigeria: primary, secondary and tertiary. Local, State and Federal Governments are responsible for primary, secondary and tertiary health care, respectively [24]. The Local and State Government Ministries of Health are responsible for coordinating health care delivery in 548 PHC facilities and 17 secondary facilities in Cross River State [19]. According to the national IPTp guidelines, health care providers are expected to administer SP at no cost to pregnant women attending ANC in public and faith-based health facilities by directly observed treatment [4].

### Sample size estimation

Using 8 % as the prevalence of SP-IPTp use at least once during antenatal care [8], this study had a power of 80 % to identify an odds ratio of 3 as significant at the 5 % level [25, 26]. The minimum sample size of 400 pregnant women was then calculated after accounting for 10 % non-response.

### Sampling technique

Multistage sampling was used to recruit 400 pregnant women attending ANC in PHC facilities. In the first stage, simple random sampling was used to select the southern senatorial district from the three senatorial districts in the state. In the second stage, Calabar south and Odukpani LGAs were randomly selected from the seven LGAs that constitute the southern senatorial district. The third stage involved the random selection of 15 out of the 55 PHC facilities in the two LGAs. Finally, the number of pregnant women recruited in each of the 15 PHC facilities was determined by proportionate sampling using a sampling fraction of 400/1539, where 400 is the minimum sample size and 1539 is the total number of pregnant women in the ANC rosters in the 15 PHC facilities (sampling frame) in August 2011, the month before the study commenced. The sampling fraction was multiplied by the number of patients in each facility-specific roster to estimate the number of patients per health facility (Additional file 1). Thereafter, pregnant women were recruited by systematic sampling until the desired sample size in each clinic was achieved. The sampling interval of 1 in 4 was used for the systematic sampling in each facility with the starting point determined by simple random sampling. The sampling interval was derived by dividing the sampling frame (1539) by the minimum sample size (400). Inclusion criteria were the permanent residency status of the prospective study participants in the community served by the PHC facilities and having been registered in the clinic roster.

### Training and quality assurance

Data were collected using a semi-structured questionnaire that gathered information on socio-demographic variables, obstetric characteristics, knowledge of malaria and malaria prevention practices for the index pregnancy (Additional file 2). Fifteen field workers and five supervisors were trained for three days on the administration of the questionnaire. The questionnaire was pre-tested in a PHC facility that was not on the list of study sites in the main study. A debriefing session was held after the pre-test to review challenges. Each field worker was assigned to a PHC facility for data collection. Each supervisor oversaw data collection in three PHC facilities. The supervisors randomly checked completed questionnaires for errors and inconsistencies immediately after the interviews. Questionnaires with errors were immediately returned to the field worker for reconciliation before data entry.

### Variables

The outcome variable for the binary logistic regression was the use of SP-IPTp in index pregnancy, with “yes” or “no” responses coded as 1 or 0 respectively. A total of 12 independent variables were analysed in the bivariate analysis. Among them were knowledge of bed nets, ITNs and SP-IPTp as means of preventing MIP along with the use of bed nets, and ITNs to prevent malaria in index pregnancy. Other independent variables were obstetric and socio-demographic characteristics of the respondents such as women in their first pregnancy, number of pregnancies, gestational age at registration for ANC in index pregnancy, education, age, occupation and socio-economic status. The socio-economic status (SES) of respondents was constructed from 22 variables on household possessions and housing characteristics using the principal components analysis (PCA) technique. [27] The respondents’ total SES scores were categorised into quintiles and labelled in ascending order: lowest; middle low; middle; middle high; and highest.

### Statistical analysis

Statistical analyses were conducted using STATA® version 12. Mean values were calculated for continuous variables while categorical variables were presented in percentages. The cut-off point for the univariate logistic regression (bivariate) analysis was set at 20 % significance level. The relationship between each of the 12 independent variables and use of SP-IPTp was analysed by forward selection in the bivariate analysis. Variables that were not significantly (confidence intervals including the null value of one) associated with use of SP-IPTp in the bivariate analysis were excluded in the final model. Interaction between independent variables was tested. Variables that were significantly (confidence intervals excluding the null value of one) associated with the use of SP-IPTp in the bivariate analysis were included in the multivariate logistic regression at 5 % significance level. In order to minimise the chances of underestimating the standard error, which arises from clustered nature of data, we declared the data as survey data and used the “svyset” command in the regression analysis. Estimates and standard errors were then adjusted for the sampling design and weighted based on the probability of a pregnant woman being selected.

### Ethical clearance

Ethical approval for this study was obtained from the Cross River State Research Ethics Committee. Permission to conduct the study was received from the nurse-in-charge of the selected PHC facilities. Field workers described the study to the pregnant women and then obtained written informed consent before enrolling the women in the study.

## Results

### Socio-demographic and obstetric characteristics of the respondents

Four hundred (400) pregnant women attending ANC in the selected PHC facilities participated in the study. Table 1 summarises the socio-demographic characteristics of the respondents. The mean age of the women was 25 ± 5.7 years, with nearly half (47 %) of the respondents in the 15–24 year age category. Table 2 shows that 80 % of the women were in their second trimester at the time of ANC registration.

**Table 1:** Socio-demographic characteristics of the respondents attending ANC in Cross River State

Variable	n (%) (95 % CI)
Age (years)	
15–24	187 (46.8) (42.8–51.8)
25–34	183 (45.7) (40.8–50.8)
35–49	30 (7.5) (5.1–10.5)
Mean (SD) (95 % CI)	25.2 (5.7) (24.7–25.8)
Education	
None	28 (7.0) (4.7–10.0)
Primary	87 (21.7) (17.8–26.1)
Secondary	226 (56.5) (51.5–61.4)
Tertiary	59 (14.8) (11.4–18.6)
Religion	
Christianity	399 (99.7) (98.6–99.9)
Islam	1 (0.3) (0.1–1.4)
Marital status	
Single	79 (19.7) (16.0–24.0)
Married	310 (77.5) (73.1–81.5)
Others^a^	11 (2.8) (1.4–4.9)
Ethnicity	
Efik	119 (29.8) (25.3–34.5)
Ibibio	145 (36.2) (31.5–41.2)
Anang	83 (20.8) (16.9–25.1)
Others^b^	53 (13.2) (10.1–17.0)
Occupation	
None	78 (19.5) (15.3–23.7)
Farming	53 (13.2) (10.1–17.0)
Trading	158 (39.5) (34.7–44.5)
Fishing	8 (2.0) (0.9–3.9)
Civil service	66 (16.5) (13.0–20.5)
Others^c^	37 (9.3) (6.6–12.5 )
Socio-economic status	
Lowest	67 (16.8) (13.7–20.1)
Middle low	91 (22.7) (19.3–26.5)
Middle	82 (20.5) (17.2–24.1)
Middle high	80 (20.0) (16.8–23.6)
Highest	80 (20.0) (16.8–23.6)

^a^Others included divorced, separated and widowed

^b^Others included other ethnic groups other than Efik, Ibiobio and Anang

^c^Others included students and artisans

**Table 2:** Obstetric characteristics of the respondents attending ANC in Cross River State

Variable	n (%) (95 % CI)
Gestational age at registration for ANC (months)	
1–3 (First trimester)	32 (8.0) (5.5–11.1)
4–6 (Second trimester)	319 (79.8) (75.5–83.6)
7–9 (Third trimester)	49 (12.2) (9.2–15.9)
Mean gestational age (SD) (95 % CI)	5 (1.2) (4.9–5.1)
Gestational age at time of interview (months)	
4–6 (Second trimester)	249 (62.2) (57.3–67.1)
7–9 (Third trimester)	151 (37.8) (33.0–42.7)
Mean gestational age (SD) (95 % CI)	6 (1.3) (5.9–6.3)
Total number of pregnancies	
1(Primigravid women)	166 (41.5) (36.6–46.5)
2–4	178 (44.5) (39.6–49.5)
≥ 5	56 (14.0) (10.8–17.8)
Total number of children	
0 (Primigravid women)	166 (41.5) (36.6–46.5)
1–4	221 (55.2) (50.2–60.1)
≥ 5	13 (3.3) (1.7–5.5)
Total number of miscarriages (n–234)	
0	158 (67.5) (61.1–73.5)
1	64 (27.4) (21.7–33.5)
2	12 (5.1) (2.7–8.8)

### Knowledge of malaria prevention practices in pregnancy

Table 3 shows that 62 % (248/400), 75 % (298/400) and 64 % (256/400) of women knew that mosquito bed nets, ITNs and SP can be used to prevent MIP, respectively. Of the 256 women who knew that SP are used for IPTp, 86 % knew the correct dose (number of pills) of SP to be taken, whereas, only 38 % knew that SP should be taken at the beginning of the second trimester.

**Table 3:** Knowledge of malaria prevention in pregnancy among respondents attending ANC in Cross River State

Variable	n (%) (95 % CI)
Mosquito bed nets	
Yes	248 (62.0) (57.0–66.8)
No	152 (38.0) (33.2–43.0)
Insecticide-treated mosquito bed nets	
Yes	298 (74.5) (70.0–78.7)
No	102 (25.5) (21.3–30.1)
SP	
Yes	256 (64.0) (59.1–68.7)
No	144 (36.0) (31.3–40.9)
Correct dose of SP (*n* = 256)	
Yes	220 (85.9) (81.1–90.0)
No	36 (14.1) (10.1–18.9)
Gestational age for taking SP (*n* = 256)	
First trimester	44 (17.2) (12.8–22.4)
Second trimester	97 (37.9) (31.9–44.1)
Third trimester	22 (8.6) (5.5–12.7)
Don’t know	93 (36.3) (30.4–42.5)

### Malaria prevention practices in index pregnancy

About 18 and 9 % of all the pregnant women, respectively, reported the use of mosquito bed nets and ITNs for malaria prevention during their index pregnancy. Approximately 41 % (*n* = 165) and 1 % (*n* = 5) of all the women, respectively, received one and two doses of SP-IPTp during the study period. Other drugs reportedly used for the prevention of malaria in the index pregnancy were chloroquine® (6 %) and antibiotics (2 %) - (Table 4).

**Table 4:** Malaria prevention practices in index pregnancy among respondents attending ANC in Cross River State

Variable	n (%) (95 % CI)
Use of mosquito bed nets	
Yes	70 (17.5) (13.9–21.6)
No	330 (82.5) (78.4–86.1)
Use of ITNs	
Yes	34 (8.5) (6.0–11.7)
No	366 (91.5) (88.3–94.0)
Use of SP	
Yes	165 (41.2) (36.4–46.2)
No	235 (58.8) (53.8–63.6)
Doses of SP received (*n* = 400)	
One	165 (41.2) (36.4–46.2)
Two	5 (1.3) (0.4–2.9)
Use of other drugs for preventing MIP	
Chloroquine®	25 (6.3) (4.1–9.1)
Antibiotics	8 (2.0) (0.9–3.9)

### Barriers to use of IPTp in index pregnancy

Table 5 shows that 83 % (*n* = 330) of the pregnant women did not have the autonomy or freedom to receive SP-IPTp during ANC without consulting a household member. Of the 165 women who utilised IPTp, 67 % (*n* = 110) received SP from the ANC clinic while 33 % (*n* = 55) received SP from a drug vendor due to drug stock-outs in the health facilities. Thirty-three percent (*n* = 36) of the 110 women who obtained SP at the ANC clinic ingested the SP by directly observed treatment.

**Table 5:** Barriers to SP-IPTp use among respondents attending ANC in Cross River State

Variable	n (%) (95 % CI)
Lack of autonomy of decision-making (*n* = 400)	
Yes	330 (82.5) (78.4–86.1)
No	70 (17.5) (13.9–21.6)
Source of SP among those who used it (*n* = 165)	
ANC clinic	110 (66.7) (58.9–73.8)
Drug vendor	55 (33.3) (26.2–41.1)
Reason for buying SP from a drug vendor (*n* = 55)	
Stock-out of SP in the ANC clinic	55 (100) (0.94–1.00)
Directly observed treatment during ANC visit (*n* = 110)	
Yes	36 (32.7) (24.1–42.3)
No	74 (67.3) (57.7–75.9)

### Determinants of the use of IPTp

In the multivariate binary regression analysis, the women who were aware that ITNs are used to prevent MIP had twofold increased odds (OR = 2.13, 95 % CI: 1.70–3.73) of using SP-IPTp compared with those with no knowledge of ITNs. Similarly, the women who knew that SP is used to prevent MIP had 22 fold increased odds (OR = 22.13, 95 % CI: 8.10–43.20) of using IPTp compared with those with no knowledge of SP. Pregnant women who used ITNs to prevent MIP had twofold increased odds (OR = 2.38, 95 % CI: 1.24–12.31) of using SP-IPTp than those who did not use ITNs (Table 6).

**Table 6:** Determinants of IPTp use among pregnant women attending ANC in Cross River State

Variable	Use of SP for the intermittent preventive treatment of malaria (*N* = 400)			
	Yes	No	Unadjusted OR	Adjusted OR
	n (%)	n (%)	(80 % CI)	(95 % CI)
	165 (41.2)	235 (58.8)		
Age group (years)				
15–24	69 (41.8)	118 (50.2)	1	^a^
25–34	81 (49.1)	102 (43.4)	1.36 (0.52–3.56)	
35–49	15 (9.1)	15 (6.4)	1.71 (0.37–7.88)	
Education				
None	7 (4.2)	21 (8.9)	1	1
Primary	31 (18.8)	56 (23.8)	1.66 (1.00–2.74)	1.72 (0.05–65.40)
Secondary	97 (58.8)	129 (54.9)	2.26 (0.62–8.22)	1.20 (0.02–61.50)
Tertiary	30 (18.2)	29 (12.4)	3.10 (2.11–4.57)	1.55 (0.03–82.20)
Occupation				
None	16 (9.7)	62 (26.4)	1	1
Civil service	37 (22.4)	29 (12.3)	0.40 (0.08–1.98)	0.75 (0.01–11.87)
Farming	18 (10.9)	35 (14.9)	0.78 (0.24–2.59)	2.72 (0.73–10.23)
Fishing	4 (2.4)	4 (1.7)	0.20 (0.04–1.17)	0.29 (0.06–13.46)
Trading	76 (46.1)	82 (34.9)	0.48 (0.07–3.42)	0.57 (0.01–16.73)
Other	14 (8.5)	23 (9.8)	0.73 (0.59–0.89)	0.88 (0.26–2.99)
Socio-economic status				
Lowest	29 (17.6)	38 (16.2)	1	1
Middle low	43 (26.1)	48 (20.4)	1.91 (1.21–3.01)	2.19 (0.06–78.02)
Middle	33 (20.0)	49 (20.9)	1.57 (0.94–2.63)	0.99 (0.10–9.91)
Middle high	36 (21.8)	44 (18.7)	2.09 (2.05–2.13)	2.82 (0.19–42.87)
Highest	24 (14.5)	56 (23.8)	1.78 (1.42–2.24)	1.24 (0.04–35.81)
First pregnancy				
No	104 (63.0)	130 (55.3)	1	^a^
Yes	61 (37.0)	105 (44.7)	0.73 (0.32–1.65)	
Number of pregnancies				
1	61 (37.0)	105 (44.7)	1	^a^
2–4	79 (47.9)	99 (42.1)	1.37 (0.61–3.08)	
≥ 5	25 (15.1)	31 (13.2)	1.39 (0.58–3.32)	
Gestational age at registration for ANC				
First trimester	16 (9.7)	16 (6.8)	1	^a^
Second trimester	132 (80.0)	187 (79.6)	0.71 (0.27–1.86)	
Third trimester	17 (10.3)	32 (13.6)	0.53 (0.21–1.37)	
Knowledge of bed nets				
No	101 (61.2)	147 (62.6)	1	^a^
Yes	64 (38.8)	88 (37.4)	1.06 (0.45–2.48)	
Knowledge of ITNs				
No	26 (15.8)	76 (32.3)	1	1
Yes	139 (84.2)	159 (67.7)	2.56 (2.28–2.87)	2.13 (1.70–3.73)
Knowledge of SP-IPTp				
No	9 (5.4)	135 (57.5)	1	1
Yes	156 (94.6)	100 (42.5)	23.4 (5.1–47.3)	22.13 (8.10–43.20)
Use of bed nets				
No	133 (80.6)	197 (83.8)	1	^a^
Yes	32 (19.4)	38 (16.2)	1.25 (0.79–1.96)	
Use of ITNs				
No	144 (87.3)	222 (94.5)	1	1
Yes	21 (12.7)	13 (5.5)	2.49 (1.83–3.39)	2.38 (1.24–12.31)

^a^Not included in the final model due to non-significance in the univariate model

Odds ratio were adjusted in the multivariate model for education, occupation, socio-economic status, knowledge of ITNs, knowledge of SP-IPTp and use of ITNs

## Discussion

The aim of this study was to identify the barriers to and determinants of the use of SP-IPTp by pregnant women attending ANC clinics in PHC facilities in Cross River State, south-south Nigeria. We observed high knowledge of the use of mosquito bed nets, ITNs and SP as means of preventing MIP. However, the actual use of these measures to prevent MIP was low. Based on the findings, we noted that the lack of autonomy or freedom to receive SP-IPTp during ANC without consulting a household member, stock-outs of free SP, and poor implementation of directly observed treatment in the ANC clinics were potential critical barriers to the use of IPTp. Knowledge and practices related to the prevention of MIP were associated with use of IPTp in the index pregnancy.

Our research findings corroborate results from similar studies conducted in PHC facilities in south-west [10, 20] and northern [14] Nigeria which showed high levels of awareness of ITNs and SP as important predictors of MIP-related preventive behaviour. The reported high knowledge of ITNs and SP as malaria preventive measures in pregnancy may be attributed to health education received during ANC, which is widely utilised by pregnant women in Nigeria [4]. The possible effects of ANC attendance on increasing awareness of malaria preventive measures in pregnancy are further corroborated by a study in Burkina Faso which showed that non-ANC users were significantly less knowledgeable about malaria/anaemia prevention measures than ANC users [28]. However, we also recognise that the knowledge gap between ANC and non-ANC users can reflect higher educational levels among women who use ANC relative to those who do not [28].

Late ANC attendance (reflected by late gestational age at registration for ANC) and poor knowledge of the gestational age for taking SP have been identified as individual barriers to the use of SP-IPTp in our study. As previously reported in Gambia [29], poor knowledge of the correct timing of ingestion of SP was associated with low use of SP-IPTp. This may have serious health implications particularly in a context where women are likely to purchase SP from service providers in the informal health sector (e.g. drug stores), some of whom may be inadequately informed about the appropriate gestational age for the ingestion of SP. As such, health education programmes targeting pregnant women and drug vendors may be needed. This is in view of the literature evidence showing that informal health care providers such as drug vendors, traditional birth attendants and adolescent peer mobilisers are capable of increasing access to and compliance with SP-IPTp [30].

The lack of autonomy or freedom to receive SP-IPTp during ANC without consultations with a family member, notably the head of the household, is a household barrier to the use of SP-IPTp in this study. Refusal to receive SP during ANC visits may be due to perceived adverse effects of SP on pregnancy as previously reported in Cross River State [15] and south-west Nigeria [10, 18]. Iliyasu et al. reported a similar finding in northern Nigeria, but attributed cultural factors as the reasons for refusal of pregnant women to use SP without prior consent from their husbands [14].

Institutional barriers to the use of SP-IPTp in this study were stock-outs of free SP due to sporadic availability of SPs in health facilities [31] and poor compliance with DOT. In assessing compliance with Nigeria’s IPTp guidelines, approximately one-third of the pregnant women who used SP-IPTp in this study were directly observed ingesting SP by a health worker. Poor compliance with national preventative treatment guidelines has been reported elsewhere in northern [14] and south-west [10, 18] Nigeria. The reasons for poor implementation of DOT in these previous studies included: (i) the practice in which pregnant women received SP from ANC clinic, but took them home in order to have a meal before taking the medicine, and (ii) procurement of SP from drug vendors, often due to drug stock-outs in the health facilities [3, 10, 14, 21]. Poor compliance with the national guidelines underscores the need to assess and enhance the capacity of PHC facilities in Nigeria to implement the DOT strategy as well as to ensure the availability of free SP in health facilities.

The 41 % prevalence of the use of one dose of SP-IPTp in this study is similar to the 40 % prevalence described by Amoran et al. [21]. The 41 % prevalence reported in our study shows an improvement over the 8 % prevalence previously reported in the 2009 NHDS [8] and 27 % prevalence described by Akinleye et al. [10] in south-west Nigeria. In Cross River State, Esu et al. reported higher (53 %) usage of one dose of SP-IPTp than the 41 % reported in this study [16]. The higher rate reported by Esu et al. can be attributed to the fact that their study was conducted in both primary and secondary health facilities with the latter recording higher ANC attendance due to the provision of ANC services therein by medical doctors. A plethora of literature showed undercoverage of SP-IPTp in Nigeria [9–16] compared with the Role Back Malaria (RBM) 80 % coverage target in 2010 [32]. Reception of the recommended minimum of two doses of SP-IPTp was very low in this study because the study participants were not followed-up till delivery due to the cross-sectional design of this study.

Knowledge of ITNs and SP as means of preventing MIP was associated with the use of SP-IPTp in this study. A similar finding was also reported in south-west Nigeria [21] and some in some sub-Saharan African countries [17]. A meta-analysis of factors affecting the use of interventions to prevent MIP showed that women who knew the benefits of SP-IPTp, and how and when to take SP were more likely to use SP-IPTp [17]. Use of ITN also determined the use of SP-IPTp, as was reported by Iliyasu et al. in northern Nigeria [14]. It is expected that mothers who use ITNs are more likely to be exposed to health education programmes focusing on the consequences of MIP and are, therefore, more willing to use SP-IPTp [14]. Similar to the findings reported by Akinyele et al. in south-west Nigeria [10], there was no significant relationship between maternal age, gestational age at registration for ANC and the use of SP-IPTp in our study.

Our study findings should be interpreted in light of the following limitations: self-reported use SP-IPTp by the women and our inability to follow-up with the women till delivery to estimate compliance with the recommended total dose of SP-IPTp according to the national guidelines. We were unable to collect facility-level data on other barriers to the use of SP-IPTp in the PHC facilities.

## Conclusions

Our study findings underscore the importance of assessing and addressing individual, household and facility factors that may impede the use of SP-IPTp. In particular, levels of awareness and non-compliance of PHC facilities to treatment guidelines may hamper efforts to reduce maternal and child morbidity and mortality associated with malaria. Health education programmes on the prevention of malaria are needed. These programmes should target mothers, heads of households and a wide range of health providers. In addition, programmes are needed to enhance the capacity of PHC facilities to implement the SP-IPTp guidelines.

### Ethics approval and consent to participate

Ethics approval for this research was granted by the Cross River State Research Ethics Committee. Permission to conduct the study was received from the nurse-in-charge of the selected PHC facilities. Written informed consent was obtained before enrolling the women to participate in the study.

### Consent for publication

Written informed consent to publish the findings of this research was obtained from the study participants. However, we did not obtain consent to publish individual person’s data.

### Availability of data and materials

The dataset on which the conclusions of this manuscript rely will not be made available given the conditions stated in the written informed consent form to protect the identity of the patients and health facilities in which the study was conducted.

## Additional files

Additional file 1: Sampling of the study participants in the PHC facilities. (DOCX 14 kb) Additional file 2: Questionnaire used for the study. (DOCX 23 kb)

## Abbreviations

ANC: Antenatal care
DOT: Directly Observed Treatment
FMOH: Federal Ministry of Health (Nigeria)
IPTp: Intermittent Preventive Treatment of malaria in Pregnancy
ITNs: Insecticide treated mosquito bed nets
LGAs: Local Government Areas
MIP: Malaria in pregnancy
NDHS: National Demographic and Health Survey
PHC: Primary Health Care
RBM: Roll Back Malaria
SP: Sulfadoxine-Pyrimethamine
SP-IPTp: Sulfadoxine-Pyrimethamine for the Intermittent Preventive Treatment of malaria in Pregnancy
SES: Socio-economic status
SSA: Sub-Saharan Africa
WHO: World Health Organization
