# Supplementary material for: Barriers to and determinants of the use of intermittent preventive treatment of malaria in pregnancy in Cross River State, Nigeria: a cross-sectional study
Source: BMC Pregnancy Childbirth. 2016 May 4;16:99. doi: 10.1186/s12884-016-0883-2 (PMC4857401; doi:10.1186/s12884-016-0883-2)
Supplement: Additional file 1: — Sampling of the study participants in the PHC facilities. (DOCX 14 kb) [file 12884_2016_883_MOESM1_ESM.docx]

Supplementary Figure 1: Sampling of the study participants in the PHC facilities

| Health facilities | Number of patients registered in the health facility roster in August 2011 | Number of patients recruited per health facility |
| --- | --- | --- |
| A | 143 | 37 |
| B | 91 | 24 |
| C | 85 | 22 |
| D | 121 | 32 |
| E | 83 | 22 |
| F | 121 | 31 |
| G | 90 | 23 |
| H | 96 | 25 |
| I | 52 | 13 |
| J | 137 | 36 |
| K | 93 | 24 |
| L | 88 | 23 |
| M | 138 | 36 |
| N | 76 | 20 |
| O | 125 | 32 |
| Total | 1539 | 400 |

**Proportionate sampling for each health facility was achieved by multiplying the sampling fraction by the total number of patients in each health facility**

Sampling fraction = 400/1539 = 0.26

Where 400 = calculated study sample size and 1539 = sampling frame

Example of proportionate sampling for clinic A: 0.26 x 143 = 37, where 143 is the total number of patients in health facility A. Same principles was applied in the proportionate sampling in the remaining health facilities.
