# Supplementary material for: Barriers to and determinants of the use of intermittent preventive treatment of malaria in pregnancy in Cross River State, Nigeria: a cross-sectional study
Source: BMC Pregnancy Childbirth. 2016 May 4;16:99. doi: 10.1186/s12884-016-0883-2 (PMC4857401; doi:10.1186/s12884-016-0883-2)
Supplement: Additional file 2: — Questionnaire used for the study. (DOCX 23 kb) [file 12884_2016_883_MOESM2_ESM.docx]

**QUESTIONNAIRE USED FOR THE STUDY**

**SECTION 1: Demographic and obstetric characteristics of respondent**

| NO | QUESTIONS | CODING CATEGORY | INSTRUCTIONS |
| --- | --- | --- | --- |
| 1 | How old are you now?  **( Age in completed years as at last birthday)** | \|  \|  \| \| --- \| --- \| |  |
| 2 | What is your ethnic origin? | Effik…………………………….…….1  Ibibio………………………………….2  Anang…………………………………3  Other (specify).……………………….4 |  |
| 3 | What is your marital status now? | Single…………………………………1  Married……………………………….2  Divorced………………………...……3  Separated……………………………..4  Widowed…………………….……….5  Other (specify)…………………….…6 |  |
| 4 | What is your level of education? | Primary………………………………..1  JSS (completed)………………………2  SSS (completed)…………….………..3  Tertiary……………………………….4  None………………………………….5 |  |
| 5 | What is your religion? | Traditional…………………………….1  Christian……………………..………..2  Muslim………………………………..3  Other (specify)………………………..4 |  |
| 6 | What is your occupation now? | None…………………………………..1  Farming..…………..………………….2  Trading..………………………………3  Fishing………………………………...4  Civil servant……………………….….5  Retired………………………………..6  Other (specify)………….…………….7 |  |
| 7 | Is this your first pregnancy? | Yes……………………………………1  No…………………………………….2 | If yes, go to 12 |
| 8 | Have you ever given birth? | Yes……………………………………1  No…………………………………….2 | If no, go to 10 |
| 9 | How many children (alive and dead) do you have? | ………..……………………………. |  |
| 10 | How many miscarriages have you had? | ………..……………………………. |  |
| 11 | How many total pregnancies have you had? (probe to ensure 11 is the sum of 9 and 10) | ……….…………………………….. | Interviewer probes |
| 12 | How many months pregnant were you when you registered for ante-natal clinic? | ……………………………………… |  |
| 13 | How many months pregnant are you now? | ……………………………………… |  |

**SECTION 2: Knowledge of how malaria is transmitted and the symptoms of malaria**

| 14 | How is malaria transmitted?  (**Circle all that apply**) | Growing teeth.………………….…..…1  Eating bad food…………………..…...2  Mosquito bites………………………...3  Eating sweet food…………………….4  Inheritance……………………………5  Standing in the sun…………………....6  Other (specify)………………………..7  Don’t Know.…………………………88 |  |
| --- | --- | --- | --- |
| 15 | How would you know that you have malaria?  (**Circle all that apply**) | Hot body………………………………1  Vomiting…..………………………….2  Body aches……………………………3  Loss of appetite……………………….4  Weakness.…………………………….5  Urine changes to yellow………………6  Shiver…………………………………7  Headache….………………………….8  Nausea………………………………..9  Dizziness…………………………….10  Don’t Know ………………………...88 |  |

**SECTION 3: Knowledge of malaria prevention in pregnancy**

| 16 | How can malaria be prevented during pregnancy?  (**Circle all that apply**) | Praying and fasting……………………1  Sleeping under insecticide treated bed .2  Sleeping under bed nets……………….3  Consulting a native doctor.....................4  Taking drugs to prevent malaria in pregnancy……………………………..5  Others (specify)……………………….6  Don’t know………………………….88 |  |
| --- | --- | --- | --- |
| 17 | Do you know what Sulfadoxine-Pyrimethamine (SPs) are?  **(Interviewer shows SPs to respondents)** | Yes……………………………………1  No…………………………………….2 | If no, go to section 4 |
| 18 | How many tablets can be taken? | …………………………………………… |  |
| 19 | What stage(s) of pregnancy can SPs be taken?  (**Circle all that apply**) | First 3 months of pregnancy………….1  Middle 3 months of pregnancy……….2  Early part of the last 3 months of pregnancy……………………………..3  Any stage of pregnancy………………4  No stage of pregnancy………………..5  Don’t know………………………….88 |  |
| 20 | Where did you get the information about the signs/symptoms from?  **(Circle all that apply**) | Radio………………………………….1  TV…………………………………….2  Community based health worker……..3  Clinic/health centre…………………...4  Hospital……………………………….5  Traditional healer……………………..6  TBA…………………………………..7  Relative/Friends………………………8  Don’t know.………………………….88 |  |

**SECTION 4: Malaria prevention practices during pregnancy**

| 21 | During this pregnancy, have you been sleeping under a mosquito bed net every night? | Yes………………………………….1  No…………………………………..2 |  |
| --- | --- | --- | --- |
| 22 | During this pregnancy, have you been sleeping under an insecticide treated mosquito bed net every night? | Yes………………………………….1  No…………………………………..2 |  |
| 23 | During this pregnancy, did you take any drugs to prevent malaria? | Yes…………………………………1  No.…………………………………2  Don’t know………………..………88 | If no go to section 5 |
| 24 | What drug(s) did you take?  (**Circle all that apply**) | SPs…………………………………1  Antibiotics…………………………2  Paracetamol………………………..3  Chloroquine…………………………4  Others……………………………….5  Don’t Know………………………..88 |  |
| 25 | If SPs were taken, how many months pregnant were you? | ……………………………..  …………………………….. |  |
| 26 | If SPs were taken, how many doses did you take? | One dose……………………………1  Two doses…………………………..2  Three doses…………………………3  More than three doses……………...4 |  |
| 27 | What was the source of the SPs? | Antenatal clinic..……………………1  Another health facility………………2  Chemist/drug vendor…...…………...3  Other source (specify)………………4  Don’t Know……………………….88 |  |
| 28 | If you bought an SP from the chemist/drug vendor, what was the main reason for purchasing the SP from the drug vendor?  (**Circle only one response**) | SPs were not available in the health facilities during antenatal care visit(s)………...……………………1  Waiting in the queues to receive SPs takes a long time…………………...2  SPs dispensed in the health facilities during antenatal care visit are substandard……..…………………3 | If the response to question 27 is Chemist/drug vendor |
| 29 | Were you directly observed swallowing an SP by a health worker during antenatal care clinic? | Yes…………………………………1  No………………………………….2 |  |
| 30 | If you received an SP during antenatal care in the health facility, did you buy the SP? | Yes…………………………………1  No………………………………….2 |  |

# Section 5: Autonomy of decision-making and advice received to prevent malaria in pregnancy

| 31 | Who decides whether you should come to the hospital for ANC?  (**Circle all that apply**) | Husband…………………..…………1  Compound head……….….…………2  Grandparents………………….…….3  Parents-in-law……………….………4  Parents…….………………….……..5  Pastor………………………………..6  TBA…………………………………7  Myself………………….………..…..8  Others (specify)……………………88 |  |
| --- | --- | --- | --- |
| 32 | Can you take drugs to prevent malaria during pregnancy without consulting someone else? | Yes………………………….………1  No…………………………….…….2 |  |
| 33 | Do you receive any form of advice from someone else to prevent malaria when you are pregnant? | Yes………………………………….1  No…………………………………..2 | If no, go to Section 6 |
| 34 | What type of advice did you receive to prevent malaria in this pregnancy?  (**Circle all that apply**) | Use Herbs………………………………..1  Consult Traditional healer..…………2  Consult Soothsayer..………………...3  See pastor…………………………...4  See TBA…………………………….5  Give drugs ………………………….6  See CHO……………………………7  Visit Health centre/clinic……………8  Visit Hospital………………………..9  Others (specify)……………………10 |  |
| 35 | Do you strictly follow the advice given? | Yes…………………………………..1  No…………………………………...2 | If no, go to section 6 |
| 36 | Who gave you the advice?  (**Circle all that apply**) | Husband…………………..…………1  Compound head……….….…………2  Grandparents………………….…….3  Parents-in-law……………….………4  Parents…….………………….……..5  Pastor………………………………..6  TBA...………………….………..…..7  Myself………………………………8  Others (specify)……………………..9 |  |
| 37 | Why do you not follow the advice given?  (**Circle all that apply**) | Old fashion advice………………….1  Wrong advice……………………….2  Expensive to use……………………3  Against my religion…………………4  Other (specify)………………………5 |  |

## Section 6: Housing characteristics and household possessions

| 1. Housing characteristics | | |  |
| --- | --- | --- | --- |
| 1.1 Does your household have a modern design?  Yes...1 No….2 | | |  |
| 1.2 What is the main material for the wall?  Mud…1 bricks…2 | | |  |
| 1.3 What is the roof of your house made of (excluding animal compounds)?  Thatch.…1 Zinc…..2 Aluminium…..3 Tiles…..4 | | |  |
| 1.4 What is the floor of your house made of (excluding animal compounds)?  Mud.…1 Concrete…..2 Tiles…..3 | | |  |
| 1.5 What are the toilet facilities for your household?  Free range…1 Pit latrine…2 KVIP…3 Pan latrine…4 WC...5 Bush……6 | | |  |
| 1.5 What is the source of the drinking water in your household?  Standing pipe…1 borehole…2 Stream…3 Well…4 | | |  |
| 2. Household possessions | |  | |
| 2.1 | How many functioning bicycles does your household have? | |  |
| 2.2 | How many functioning motorcycles does your household have? | |  |
| 2.3 | How many functioning vehicles does your household have? | |  |
| 2.4 | How many functioning radio sets does your household have? | |  |
| 2.5 | How many functioning TV sets does your household have? | |  |
| 2.6 | How many functioning sewing machines does your household have? | |  |
| 2.7 | How many functioning kerosene stoves does your household have? | |  |
| 2.8 | How many functioning electric cookers does your household have? | |  |
| 2.9 | How many functioning gas cookers does your household have? | |  |
| 2.10 | How many functioning refrigerators does your household have | |  |
| 2.11 | How many functioning fans does your household have | |  |
| 2.12 | How many functioning air conditioners does your household have | |  |
| 2.13 | How many functioning DVD/VHS players does your household have? | |  |
| 2.14 | How many sheep does your household keep? | |  |
| 2.15 | How many goats does your household keep? | |  |
| 2.16 | How many birds (chicken) does your household keep? | |  |
